# The transcription factor *Maf-S* regulates metabolic resistance to insecticides in the malaria vector *Anopheles gambiae*

**DOI:** 10.1186/s12864-017-4086-7

**Published:** 2017-08-30

**Authors:** Victoria A. Ingham, Patricia Pignatelli, Jonathan D. Moore, Simon Wagstaff, Hilary Ranson

**Affiliations:** 1 Liverpool School of Tropical Medicine, Pembroke Place, Liverpool, England L35QA; 2 Earlham Institute, Norwich Research Park Innovation Centre, Colney Lane, Norwich, England NR4 7UH

**Keywords:** Insecticide resistance, Transcriptional control, Cross resistance, Metabolic resistance, Mosquito, *Anopheles gambiae*

## Abstract

**Background:**

Malaria control in Africa is dependent upon the use insecticides but intensive use of a limited number of chemicals has led to resistance in mosquito populations. Increased production of enzymes that detoxify insecticides is one of the most potent resistance mechanisms. Several metabolic enzymes have been implicated in insecticide resistance but the processes controlling their expression have remained largely elusive.

**Results:**

Here, we show that the transcription factor *Maf-S* regulates expression of multiple detoxification genes, including the key insecticide metabolisers *CYP6M2* and *GSTD1* in the African malaria vector *Anopheles gambiae*. Attenuation of this transcription factor through RNAi induced knockdown reduced transcript levels of these effectors and significantly increased mortality after exposure to the pyrethroid insecticides and DDT (permethrin: 9.2% to 19.2% (*p* = 0.015), deltamethrin: 3.9% to 21.6% (*p* = 0.036) and DDT: 1% to 11.7% (p = <0.01), whilst dramatically decreasing mortality induced by the organophosphate malathion (79.6% to 8.0% (p = <0.01)). Additional genes regulated by *Maf-S* were also identified providing new insight into the role of this transcription factor in insects.

**Conclusion:**

*Maf-S* is a key regulator of detoxification genes in *Anopheles* mosquitoes. Disrupting this transcription factor has opposing effects on the mosquito’s response to different insecticide classes providing a mechanistic explanation to the negative cross resistance that has been reported between pyrethroids and organophosphates.

**Electronic supplementary material:**

The online version of this article (10.1186/s12864-017-4086-7) contains supplementary material, which is available to authorized users.

## Background

Insecticides play a key role in controlling malaria vectors and hence preventing disease transmission. An increase in the use of insecticide treated bednets (ITNs) and indoor residual spraying (IRS) has led to a dramatic decrease in malaria cases since 2000 [1]. However, increased exposure to a limited range of insecticides has led to the emergence of resistance to these compounds [2] which poses a serious threat to the future of malaria control efforts. Four major insecticide classes are used to control adult mosquitoes; pyrethroids, DDT, carbamates and organophosphates. All four are used in IRS programmes but all ITNs are treated with pyrethroids [1]. Resistance to all classes has been reported in African malaria vectors with pyrethroid resistance spreading exceptionally rapidly in recent years [2]. Understanding the mechanisms underpinning this resistance is an important prerequisite for the identification of resistance management strategies.

The two best characterised causes of insecticide resistance are target-site resistance and metabolic resistance [3]. Mutations in the voltage-gated sodium channel, the target of both DDT and pyrethroid insecticides, cause the phenotype known as knockdown resistance or *kdr*. Although this resistance mechanism is readily tracked through PCR and therefore widely reported, metabolic resistance has a greater operational impact on malaria control [4]. Metabolic resistance is more complex to elucidate at the molecular level and can involve changes in the rate of sequestration, detoxification and/or transport of insecticides or their conjugates. Increased expression of genes involved in each of the detoxification stages has been associated with insecticide resistance in *Anopheles* mosquitoes [5] but the mechanisms controlling expression of these genes have not been identified.

The transcriptional response to exposure and adaptation to xenobiotics is well studied in mammalian systems and is regulated by three major families, all of which cause induction of phase I, II and III drug metabolism enzymes when activated:, (i) aryl hydrocarbon receptor-aryl hydrocarbon receptor nuclear translocator (*ARNT-AhR*) [6] (ii) the pregnane X and constitutive androstane receptors (PXR and *CAR*) [7] and (iii) (i) *Nrf2-Maf* [8]. Homologs of these transcriptional regulators have been identified in invertebrates and shown to regulate genes involved in response to xenobiotics. For example, *Tango-spineless* the homologs of *ARNT-AhR,* enhance the expression of the cytochrome P450 *CYP6B1* from black swallowtail caterpillars in response to allelochemicals [9]*. DHR96,* the homolog of the human *PXR-CAR* receptors, regulates sensitivity to DDT through changes to the expression of the *CYP6* family of P450s and other key detoxification families in *Drosophila* [10]. Lastly the invertebrate equivalent of the *Nrf2-Maf* pathway in *Drosophila*, *cnc-Maf-S*, has been shown to modify resistance to both malathion and DDT when constitutively activated, through regulation of *CYP6* genes and the glutathione transferase *GSTD* family [11, 12]. The *cnc-Maf-S* pathway has also been implicated in the regulation of the *CYP* families in the greenfly, *Aphis gossypii* [13] and beetles *Tribolium castaneum* and *Leptinotarsa decemlineata* [14, 15].

**Fig. 1:**
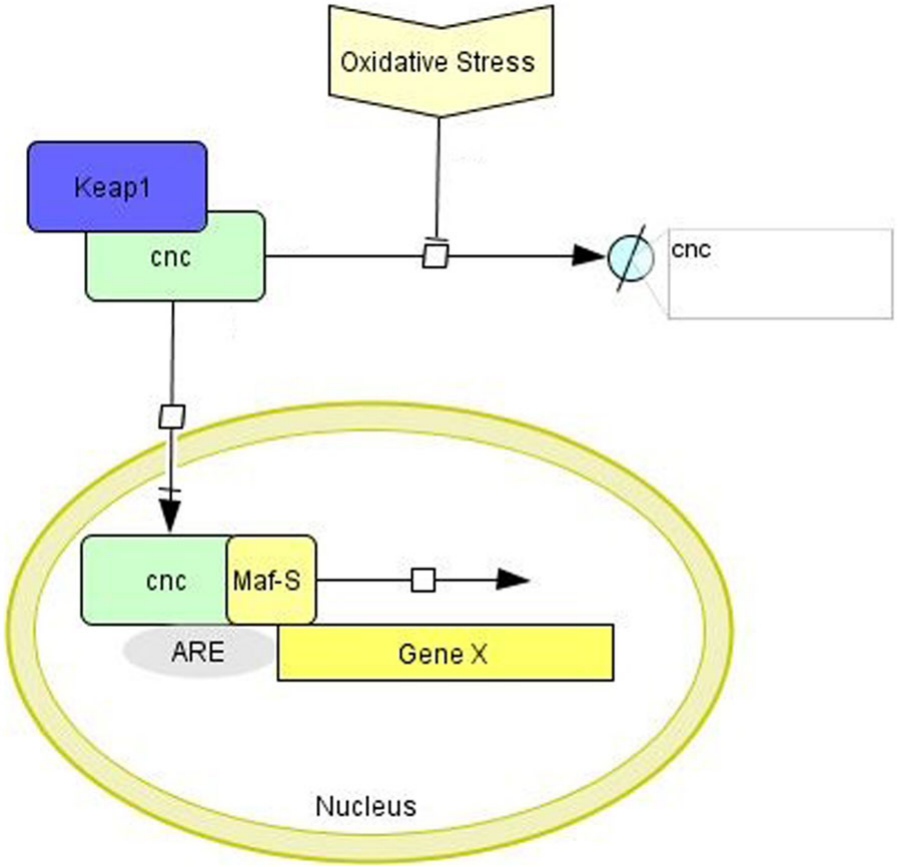
*Maf-S-cnc-Keap1* pathway. The binding of the ubiquitin ligase *Keap1* to *cnc* results in proteasomal degradation of *cnc* in the absence of oxidative stress. Under oxidative stress conditions, proteolysis is blocked and *cnc* is released by *Keap1,* which translocates into the nucleus and binds to *Maf-S.* The *cnc-Maf-S* complex then binds to antioxidant response elements (ARE) in the genome, initiating transcription. Adapted from [30]

*Maf-S* is a small nuclear-located transcription factor that binds to antioxidant response elements (ARE) in the genome that control many genes involved in xenobiotic defence. Activation of transcription is regulated through heterodimerisation with a normally cytoplasmic protein, *cnc*. The formation of *Maf-S-cnc* complexes is in turn controlled by an actin binding ubiquitin ligase, *Keap1*. In the absence of electrophiles and reactive oxygen species, *Keap1* binds to cytoplasmic *cnc* targeting it for proteolysis [16]. Under conditions of stress, the interaction with *Keap1* is disrupted, *cnc* translocates to the nucleus, binds to *Maf-S* and activates ARE (Fig. 1). Genes putatively encoding the three components of this regulatory complex were identified in the genome of *Anopheles gambiae*, the major African malaria vector, by homology searches and examined for the presence of the functional domains: *Maf-S* is encoded by AGAP01045, *cnc* by AGAP005300 and *Keap1* by AGAP003645. Further information on each of these genes is provided in Additional file 1.

In this study, we show that *Maf-S* expression correlates with expression of multiple insecticide resistance candidates in the major malaria vector *Anopheles gambiae*, suggestive of a regulatory role for this transcription factor in insecticide resistance. This was confirmed by RNAi mediated gene knockdown which resulted in an increase in the mosquitoes’ susceptibility to pyrethroid and DDT insecticides but a decrease in their susceptibility to the organophosphate malathion. We hypothesise that this dichotomous response is mediated by one or more P450s, regulated by *Maf-S*, which are capable of both detoxifying and activating different insecticide classes.

## Results

### Mining of transcriptomes from pyrethroid resistant populations to identify genes regulated by *Maf-S*

*Anopheles gambiae* is a species complex of eight different species with three species, *Anopheles gambiae s.s., Anopheles coluzzii* and *Anopheles arabiensis* being the most important in terms of malaria transmission [17]. Pyrethroid resistance is now widespread in all three of these species [2] and previous studies to identify the molecular basis of this resistance have generated extensive datasets comparing expression of the entire transcriptome in insecticide susceptible or resistant populations (e.g [18–22]). These data sets have been mined individually to identify candidate effector genes responsible for the resistance phenotype (see references in Additional file 2: Table S1), several of which have been functionally validated [23–26]. In this study, we used these datasets to identify genes that were co-regulated with the *Maf-S* transcription factor. As variable study designs have been employed in the microarray experiments, we filtered the datasets to only consider experiments where gene expression was compared between a field collected pyrethroid resistant population and a standard susceptible population. A total of 27 data sets meeting the above criteria were identified (Additional file 2: Table S1).

The 27 microarray data sets were analysed using limma [27] to identify transcripts whose expression was strongly correlated with *Maf-S* and co-factors, through use of correlation networks. Fourteen transcripts were strongly correlated (>0.8) with *Maf-S* expression (Additional file 3: Figure S2 and Additional file 4: Table S2) including two cytochrome P450s (*CYP6M2* and *CYP4H17*) and two glutathione transferases (*GSTD1* and *GSTD3*).

Expression of *cnc* is correlated with 254 transcripts; GO term enrichments of these transcripts demonstrated roles in signalling, development and regulation and transcriptional cofactor activity, consistent with a role in transcriptional activation (*p* ≤ 0.05) (Additional file 4: Table S2). *Keap1* had just three co-correlated transcripts, including *TPX4*, a juvenile hormone inducible protein and a GTPase (Additional file 4: Table S2). There was no overlap between the genes co-regulated with *Maf-S*, *cnc* or *Keap1* in the insecticide resistance microarray data sets (Additional file 4: Table S2) nor is there any evidence of co-regulation of these three transcripts in the larger data set of microarray experiments available on VectorBase [28]

### Maf-S knockdown

To test whether *Maf-S* regulates expression of the pyrethroid resistance-associated transcripts, *Maf-S* levels were reduced via RNAi and then expression of transcripts encoding detoxification enzymes present in the correlation networks were quantified by qRT-PCR. Mosquitoes were first injected with two separate dsRNAs for *Maf-S* and with *dsGFP* used as a control. The degree of knockdown was determined by qPCR comparing *Maf-S* levels in *GFP* injected mosquitoes with either of the two *Maf-S* dsRNAs. The dsRNA with the most efficient knockdown of *Maf-S, Maf-S (2),* was used in all further experiments (ratio of *Maf-*S (1): 0.56 and *Maf-S* (2)*;* 0.26 compared to *GFP*-injected controls) (Additional file 5: Figure S3).

**Fig. 2:**
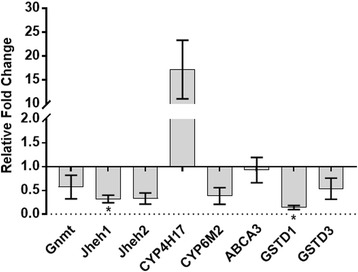
Gene expression in *Maf-S* knockdowns relative to GFP injected controls. qPCR of three transcripts regulated by the *cnc-Maf-*S pathway in *Drosophila* (*Gnmt*, *Jheh1*, *Jheh2*) and five selected from the *Maf-S* co correlated genes in *Anopheles* (*CYP4H17*, *GSTD3*, *ABCA3*, *CYP6M2, GSTD1*) was performed on *Maf-S* knockdown cDNA. Data are shown normalised against expression in GFP-injected controls. Significance (*p* ≤ 0.05), as determined by a Welch’s t-test, is indicated by an *. Each data point represents the mean of three biological replicates each comprising cDNA from 7 to 10 females, 72 h post injection (actual age of mosquitoes = 7–8 days)

Next, the expression of eight transcripts, five identified via the correlation network and three inferred from previous publications [11], were compared in mosquitoes injected with dsRNA against *Maf-S* compared to *GFP*-injected and uninjected controls. The eight genes comprised (i) four detoxification predicted by our correlation network to be co-regulated with *Maf-S* in *An. gambiae* (*GSTD1* (AGAP004164)*, GSTD3* (AGAP004382)*, CYP6M2* (AGAP008212) and *CYP4H17* (AGAP008358)), (ii) an ABC transporter *ABCA3* (AGAP007504) also identified via the correlation network, (iii) orthologues of three genes shown to be regulated by the *Maf-S-cnc-Keap1* pathway in *D. melanogaster* (two orthologs of the juvenile hormone epoxide hydrolases (*Jheh*: AGAP008684 and AGAP008685) and a glycine N-methyl transferase (*gnmt*, AGAP002198)) [11]. Suppressing expression of *Maf-S* resulted in reduced expression of six of the eight transcripts tested, two significantly (*GSTD1* and *Jheh1)*. Three of the four detoxification genes, *CYP6M2*, *GSTD1* and *GSTD3,* were reduced as predicted by the correlation network whereas *CYP4H17* showed elevated expression when compared to the GFP injected controls (Fig. 2). The correlation networks were not predictive of expression of the ABC transporter *ABCA3,* which showed no clear change in expression after knock-down. All three of the transcripts selected from the genes regulated by *Maf-S* in *Drosophila* showed reduced expression in *Maf-S* knocked down groups suggesting that these genes are also regulated by the *Maf-S* pathway in mosquitoes (Fig. 2).

The up- and down-stream sequences from the annotated gene sequences were examined to identify potential motifs that may be involved in *Maf-S* transcription. The antioxidant response element (ARE) motif was first described in the mammalian system with a consensus motif 5′-TMAnnRTGAYnnGCRwwww-3′ [29] and later identified downstream of *GSTD1* in *Drosophila* in the same form [30]; in both cases the ARE was shown to be the binding site of *cnc-Maf-S*. The presence of the *Drosophila* ARE motif, from JASPAR CORE Insect [31] (Additional file 6: Figure S4), was determined in 2000 base pairs upstream and downstream from each of the *Maf-S* co-correlated transcripts, using a motif searching package on R [32]. Twelve of the 14 transcripts identified by *Maf-S* correlation network analysis contained the ARE motif, including *Maf-S* itself, with 9 containing either an upstream or downstream ARE motif and a further two, *GSTD3* and AGAP006662, containing both up and downstream ARE motifs (Additional file 6: Figure S4). ARE binding sites were not detected in *ABCA3* or *CYP4H17*, which is consistent with the observation that expression of these genes were not suppressed following knock-down of *Maf-S* (Fig. 2) but were detected in the flanking regions of the three Drosophila orthologs which we suppressed by *Maf-S* knockdown. Interestingly, *cnc, Keap1* and *Maf-S* each contain up- and downstream occurrences of the ARE motif, which may be indicative of an auto-regulatory loop, similar to that described in mammalian systems and in *Drosophila* [30, 33, 34].

Having confirmed that *Maf-S* regulates expression of key detoxification genes in *An. gambiae*, we sought to obtain a more comprehensive picture of the genes controlled by this transcription factor by microarray analysis. RNA was extracted from the Tiassalé strain of *An gambiae* [35], 72 h post injection of *Maf-S(2)* or *GFP* dsRNA and competitively hybridised to an *An. gambiae* microarray [23]. A total of 3401 transcripts were significantly differentially expressed (*p* ≤ 0.05), of these 1703 were down-regulated in the *Maf-S* silenced group and 1698 were up regulated (Additional file 7: Table S3 and Additional file 8: Figure S5). The up-regulated transcript list is highly enriched in ion transport, ATP and purine processes and ligand channel activity whereas the down-regulated transcript list shows enrichment in key terms such as RNA binding, ribonucleoprotein and cytosol (Additional file 9: Table S4). Whilst it is recognised that the differentially expressed transcripts in the microarray experiment may include genes not directly regulated by *Maf-S,* it was encouraging that three of the six genes (*GSTD1*, *CYP6M2*, *Gnmt)* previously shown to be regulated by *Maf-S* by qPCR were also significantly down regulated in the *Maf-S* silenced mosquitoes in the microarray dataset.

The differentially expressed genes included 64 transcripts from detoxification families commonly associated with insecticide resistance (glutathione transferases (GSTs), cytochrome P450s, carboxylesterases (COEs), UDP-glucuronosyltransferases (UGTs) and ABC transporters), 30 in the up regulated list and 34 in the down regulated list (Additional file 10: Table S5). The genes, down-regulated after *Maf-S* silencing, included 17 P450s, two ABC transporters, eight GSTs, four UGTs and three COEs (Additional file 10: Table S5 and Additional file 8: Figure S5). In addition to *CYP6M2* the down-regulated P450s included *CYP6Z2*, *CYP6Z3*, *CYP6P4, CYP4G16* and *CYP4G17* and GSTD1, all of which have been associated with insecticide resistance in *An. gambiae* [36]. Two further cytochrome P450s linked to ecdysone biosynthesis were also down –regulated by *Maf-S* silencing: *CYP302A1* and *CYP314A1* [37]. Detoxification genes with higher numbers of transcripts following *Maf-S* silencing included five ABCs, six COEs, 15 cytochrome P450s, two GSTs and two UGTs. Of these only *CYP9K1* is a strong candidate for conferring insecticide resistance in *An. gambiae* [38].

### Maf-S regulates the response to insecticides in anopheles

In *Drosophila* the *Maf-S-cnc-Keap1* pathway has been shown to play a role in resistance to both malathion and DDT [12]. In order to evaluate the role of this pathway in insecticide resistance in *An. gambiae*, *Maf-S* knockdowns were again produced by RNAi experiments in the Tiassalé strain*.* This strain, originally colonised from rice fields in southern Cote d’Ivoire [35] is resistant to DDT, carbamates and pyrethroids but susceptible to the organophosphate malathion according to World Health Organisation definitions [39]. *Maf-S* knocked down Tiassalé mosquitoes were exposed to insecticides 72 h after RNAi injection and mortality recorded a further 24 h later. The discriminating doses set by WHO were used for all insecticides and the standard 60 min exposure used for the pyrethroids, bendiocarb and DDT but, as the uninjected controls were all killed by 60 min exposure to the discriminating dose of malathion, shorter exposure times of 5 min were used to detect the effect of *Maf-S* silencing on insecticide susceptibility.

**Fig. 3:**
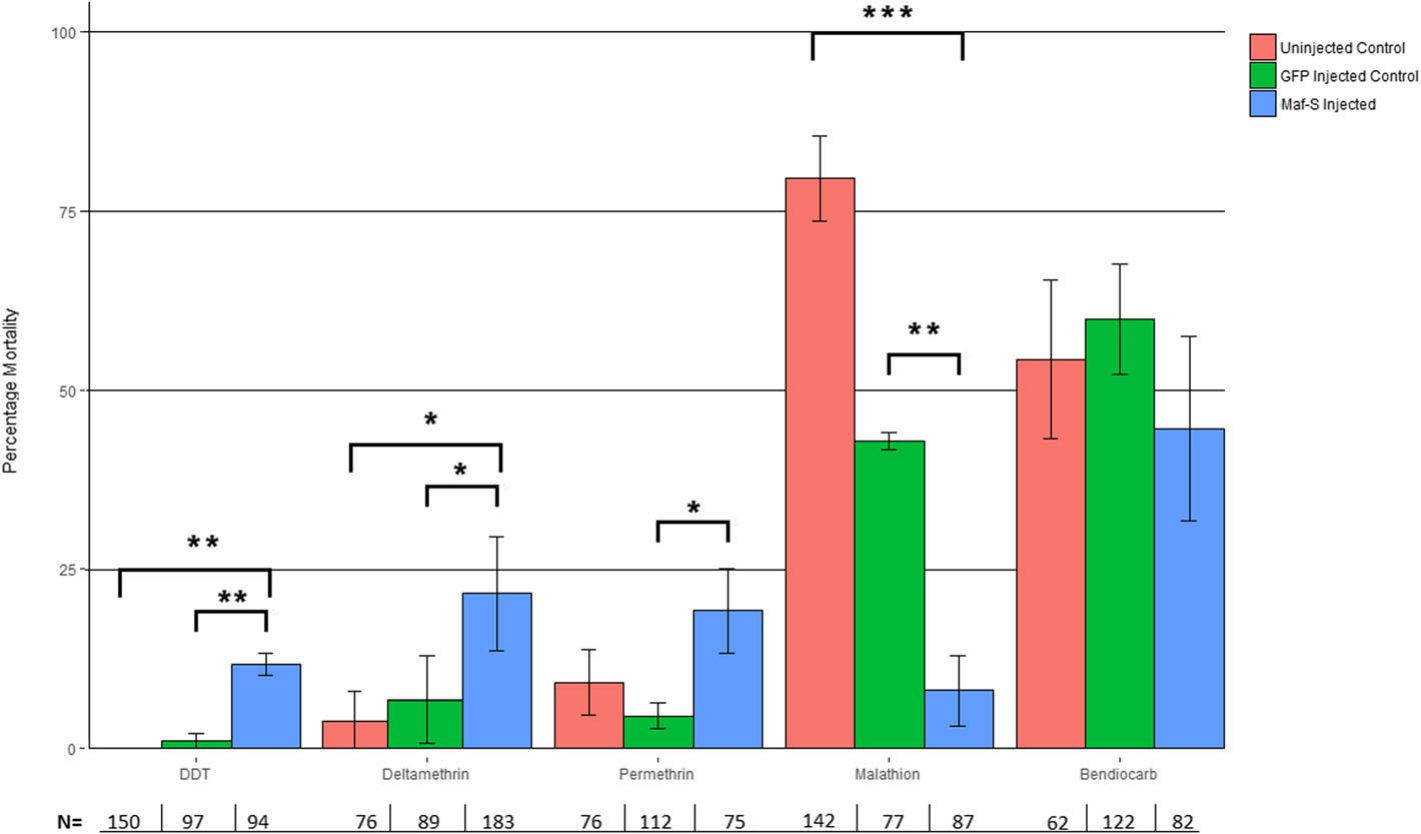
Insecticide bioassays on *Maf-S* silenced adults. *An. gambiae sl* female mosquitoes were injected with *Maf-S* or *GFP* dsRNA*,* and then exposed to insecticides using the WHO tube assay. Exposure times were 60 min to papers coated with 4% DDT, 0.75% permethrin, 0.05% deltamethrin or 0.1% bendiocarb; and 5-min to 5% malathion papers. Significance is represented by: * *p* ≤ 0.05, ** *p* ≤ 0.01 and *** *p* ≤ 0.001 displayed above each bar, as calculated by ANOVA with a Tukey post hoc test. Error bars represent standard error. Number of mosquitoes per test for each insecticide/condition are represented by numbers below the x-axis. Each WHO test had 20–25 female mosquitoes per tube

Silencing *Maf-S* significantly increased mortality (i.e. reduced the resistance) of the Tiassalé strain to DDT (p = <0.01), and the pyrethroids deltamethrin and permethrin (*p* = 0.036 and *p* = 0.015, respectively) but had no impact on bendiocarb induced mortality (Fig. 3). In contrast, silencing *Maf-S* decreased the susceptibility of the mosquitoes to malathion (increased their resistance) (p = <0.01). Unexpectedly, mosquitoes injected with *dsGFP* also showed lower mortality after malathion exposure than the uninjected controls, nevertheless, there was a significantly reduced mortality in the *Maf-S* versus *GFP* injected groups (*p* = 0.0054). Malathion is a pro-insecticide, which is activated to the insecticidal oxon form by P450s. The observation that supressing the *cnc-Maf-S* pathway increases pyrethroid toxicity but decreases malathion toxicity in vivo suggests that constitutive elevation of this pathway could result in resistance to some insecticide classes whilst simultaneously increasing the susceptibility to other insecticide classes. This ‘negative cross resistance’ has major operational implications for malaria control, as discussed below.

## Discussion

Significant progress has been made in recent years in identifying the metabolic enzymes responsible for elevated detoxification in insecticide resistant pest species [40]. However, the central question of the mechanisms underpinning increased gene expression are poorly understood. Copy number variation has been implicated in elevated P450 levels in some insecticide resistant populations but in most cases, even where gene duplication does occur, transcriptional regulation is thought to be involved [41, 42]. The *cnc*-*Maf-S* pathway has recently been shown to be conserved and constitutively activated in DDT- and malathion-resistant *Drosophila melanogaster* [11, 12]*,* deltamethrin-resistant red flour beetles [14] and imidacloprid-resistant Colorado potato beetles [15]. The initial objective of this study was to determine whether the same pathway was also active in pyrethroid resistant populations of malaria vectors. To do this we took advantage of previously generated datasets comparing transcriptomes of pyrethroid resistant and susceptible *An. gambiae* populations and used a bioinformatics approach to identify genes that appeared to be co-regulated with the three components of the complex. We identified a large number of genes whose expression was correlated with *cnc* transcript levels, several of which may be related to the multiple roles of *cnc* in homeostasis, development, metabolism and aging [43]. A much smaller number of genes (14) were identified in the correlation networks of *Maf-S* and nearly half of these belonged to four gene families typically implicated in insecticide metabolism and transport.

Next, we used RNAi to knockdown expression of *Maf-S* and confirmed that transcripts of a subset of these co-regulated genes were also depleted in the *Maf-S* knockdowns. Included amongst these genes were *CYP6M2,* a proven pyrethroid metaboliser [26] and *GSTD1*, which catalyses the dehydrochlorination of DDT [25]. Subsequent microarray experiments also identified further detoxification gene transcripts suppressed by *Maf-S* silencing including*, CYP6Z2, CYP6Z3, CYP4G16, CYP4G17,* and *CYP6P4* all previously linked to pyrethroid and/or DDT resistance [18–22, 36]. Although a similar number of detoxification genes were found to have elevated expression in the *Maf-S* silenced mosquitoes, this subset of genes does not contain any with known affinity for binding and/or metabolising insecticides and expression of only one of these, *CYP9K1*, has been associated with insecticide resistance [38]. Although the ARE motif is present downstream of *CYP6P3* a pyrethroid metaboliser associated with pyrethroid resistance in multiple populations [24], this gene was not significantly differentially expressed in the microarray data set after *Maf-S* knockdown; this may indicate that *CYP6P3* is regulated independently of this pathway although it should also be noted that the microarray experiments did not detect all of the genes we confirmed to be regulated by *Maf-S* by qPCR.

Enrichment of *Maf-S* expression in the midgut and malpighian tubules [36, 44] (tissues implicated in detoxification in insects [37, 45]), and the importance of this transcription factor in controlling expression of genes encoding known insecticide metabolising enzymes, were supportive of a role in insecticide resistance [36]. This was confirmed by insecticide bioassays which showed that supressing expression of *Maf-S* resulted in an increase in susceptibility to the pyrethroids deltamethrin and permethrin, and the organochlorine DDT, in the Tiassalé strain which is normally highly resistant strain to these insecticide classes [35, 46]. We note that susceptibility was not fully restored after *Maf-S* silencing; this was not unexpected as the Tiassalé strain is known to contain additional resistance mechanisms, such as target site mutations, that are not regulated by *Maf-S*. Furthermore, injection of dsRNA did not fully silence expression of this gene, and this coupled with the unknown turnover rates for detoxification proteins, may have resulted in continued expression of *Maf-S* regulated genes, all be it at a reduced level.

Previous microarray studies have identified a subset of P450 genes that are elevated in the Tiassalé resistant strain compared to susceptible populations [20]; these include *CYP6M2, CYP6P3, CYP6P4, CYP6Z2* and *CYP6Z3*. We show that all of these P450s, with the exception of *CYP6P3*, are regulated by *Maf-S*; furthermore several of these P450s have been shown to metabolise pyrethroids and/or DDT via in vitro characterisation of recombinant enzymes (26 and our unpublished data). Our data therefore suggests that depletion of *Maf-S* reduces levels of key pyrethroid and DDT detoxifying enzymes and increases the susceptibility of the resistant strain to these chemicals. Other detoxification genes regulated by *Maf-S*, but with as yet uncharacterised roles in insecticide metabolism, are also over expressed in the Tiassalé resistant strain (see Additional file 10: Table S5) and these warrant further investigation.

The *cnc*-*Maf-S* pathway in invertebrates has multiple functions in addition to its role in mediating response to oxidative stress and xenobiotics. For example the *cnc*-*Maf-S* heterodimer is involved in *Drosophila* development [16] and control of energy metabolism [47, 48]. Furthermore, in *Drosophila, cnc-Maf-S* plays a part in ecdysone biosynthesis [49]. Ecdysteroids determine moulting timing, and their precursors are modified by a subset of cytochrome P450s known as the Halloween genes*:* Phantom (*CYP306A1*), disembodied (*CYP302A1*), shadow (*CYP315A1*) and shade (*CYP314A1*). Of these genes, *CYP302A1* and *CYP314A1* were both down regulated after *Maf-S* silencing in this study. Furthermore, two juvenile hormone esterase hydrolases which catalyse the enzymatic degradation of juvenile hormone [50] are also regulated by *Maf-S* in *Anopheles* and *Drosophila* [11,12, this study] supporting a role for this pathway in metamorphosis.

### An unexpected impact of disruption of the Maf-S pathway on malathion resistance.

Whilst attenuating the *Maf-S* pathway decreased the mosquitoes’ ability to withstand DDT and pyrethroid exposure, it had a dramatic opposing effect on the response to malathion exposure. The Tiassalé strain of mosquitoes is highly susceptible to malathion (exposure to the discriminating dose for 60 min resulted in 100% mortality). Surprisingly however, when mosquitoes were exposed for 5 min, <10% in the *Maf-S* silenced population were killed compared to 80% of the uninjected control and 43% of the GFP control. Malathion is a pro-insecticide which is activated to the much more toxic oxon form in the insect; this oxidative desulfuration reaction is catalysed by P450s [51]. As described above, *CYP6M2*, along with members of the CYP6P and CYP6Z family are the most highly upregulated P450s in the insecticide resistant Tiassalé strain [52]. *CYP6M2*, has a high affinity for malathion and the primary metabolite after incubating malathion with recombinant *CYP6M2* has a molecular mass of 315, consistent with the activated form malaoxon [53]. Thus, the decreased malathion induced toxicity observed could be explained by a reduction in *CYP6M2* levels in the *Maf-S* knockdowns, as confirmed in both the array and qPCR results, reducing the rate of activation of malathion to malaoxon and reducing the toxic effect of the insecticide in these knockdown mosquitoes.

Interestingly, the opposite phenotype was observed in *D. melanogaster*. Here activating the *Maf-S* pathway*,* by depletion of the repressor *Keap1,* resulted in increased resistance to malathion [12] suggesting that the enzymes responsible for malathion detoxification, and not activation, are regulated by *Maf-S* in this species. Clear 1:1 orthology is rare in the *Anopheles* and *Drosophila* P450 families [54] and as far as we are aware, the P450s activating malathion in *Drosophila* have not been identified and thus it is not yet possible to validate this prediction. However, the finding that perturbing a single transcription factor in *Anopheles* can increase susceptibility to one insecticide class whilst simultaneously reducing the susceptibility to a chemically unrelated class provides important insights into metabolic resistance to insecticides which may have operational implications for resistance management.

Experimental hut studies from Côte d’Ivoire found malathion to be highly effective against the pyrethroid resistant Tiassalé population and it has been postulated that ‘negative cross resistance’ may be enhancing the efficacy of organophosphates in this setting [55]. Negative cross resistance between pyrethroids and pro-insecticides, mediated by elevated cytochrome P450 activity in the insects, has been demonstrated in agricultural pests [56] but has so far remained a largely untested theoretical hypothesis in mosquitoes. Here we provide evidence for its existence, and identify both an effector enzyme and regulatory pathway. *CYP6M2* is up-regulated in the Tiassalé population in Côte d’Ivoire [52] and thus this P450 (and/or possibly others regulated by the *cnc-Maf-S*) pathway could both enhance the efficacy of pyrethroids whilst also increasing the potency of malathion (and potentially other pro-insecticides).

## Conclusion

This study provides novel insights into the transcriptional regulation of insecticide resistance in the malaria vector, *An. gambiae*. We demonstrate that the transcription factor *Maf-S* controls expression of multiple detoxification-related transcripts, including the proven pyrethroid and DDT metabolisers, *CYP6M2* and *GSTD1*. Secondly we show that silencing expression of *Maf-S* leads to increased susceptibility to these insecticides. Finally, we provide evidence that the *Maf-S* pathway regulates one or more P450s responsible for the negative cross-resistance between pyrethroids and organophosphates. Malaria endemic countries are facing a public health crisis as resistance erodes the efficacy of the limited chemicals available to target the mosquito vectors [57]. The results from this study highlight the value of understanding the molecular basis of resistance and offer hope that this information can be used to introduce effective strategies to manage resistance.

## Methods

### Microarray analysis

Microarray datasets listed in (Additional file 2: Table S1), and associated metadata were provided by members of the Department of Vector Biology at LSTM and are publicly accessible in ArrayExpress (Accession numbers listed in Additional file 2), VectorBase or through the respective publications. All microarray datasets were analysed using the base limma R package [58], applying linear models to correct and normalise data, inferring differential transcript expression. Data was normalised using affycoretools [59]. Both within (loess) and between (aquantile) arrays, in addition to background correction (mle) were performed. Dye swap correction and design matrices were used where necessary. False discovery rate testing was used for multiple test correction. All other parameters were kept as default. All information for use can be found on Bioconductor (https://bioconductor.org/packages/release/bioc/html/limma.html).

### Probability of a transcript being differentially regulated in n arrays by chance

Each of the microarray datasets listed in Additional file: Table S1 were used to calculate the probabilities of success (significance) and failure (non-significance) of a given transcript. The probability of success was calculated using the average number of all significant transcripts as a proportion of the overall number of transcripts on the array. The probability of success, over all arrays, was 0.4692 and that of failure, 0.5308. These data were used to calculate whether each transcript in the *Maf-S-cnc* pathway was significant in more data sets than expected by chance.

### Enrichment tests

Enrichments of transcript lists for both correlation networks and microarray data were performed using the DAVID functional annotation tool (https://david.ncifcrf.gov/summary.jsp) [60] for transcript lists that were longer than 100 transcripts. Smaller transcript lists were assessed for specific enrichments using a hypergeometric test in R. In both cases, significance was determined as *p* ≤ 0.05.

### Correlation networks

Correlation networks were produced using a correlation matrix with a Euclidean distance metric produced in R, with fold changes of all transcripts from all 27 available microarrays (Additional file 2: Table S1). To identify only genes with a strong correlation or anti-correlation, only those transcripts with a correlation of ±0.8 were used. These data were extracted from the correlation matrix along with associated log_2_ fold change for each of the separate microarray experiments and viewed in Cytoscape [61] as a biological network.

### Mosquito rearing conditions

The *An. gambiae* s.l used in these experiments were from the Tiassalé strain originally from Côte D’Ivoire but maintained under pyrethroid selection pressure in the insectaries at the Liverpool School of Tropical Medicine since 2013. This strain is resistant to pyrethroids and DDT but susceptible to malathion [35, 52] (Fig. 3). Mosquitoes were reared under standard insectary conditions at 27 °C and 70–80% humidity under a 12:12 h photoperiod.

### RNAi

RNAi constructs were produced for *Maf-S* in the form of dsRNA for microinjections (Additional file 11: Table S6). PCR was performed using Phusion® High-Fidelity DNA Polymerase (Thermo Scientific) following manufacturer’s instructions and primer sets with a T7 docking sequence at the 5′ end of both the sense and antisense primers (Additional file 11: Table S6). Primers were designed to produce an asymmetric product with a length of 300-600 bp, a GC content of 20–50% and no more than three consecutive equivalent nucleotides. PCR was performed with the following cycle: three minutes 98^o^c, 35 cycles of seven seconds at 98^o^c and 10 s at 72^o^c, with a final hold at 72^o^c for seven minutes. PCR products were resolved on 1% agarose gels for 45 min and the correct length amplifications identified. The PCR products were purified using a Qiagen QIAquick PCR Purification kit following manufacturer’s instructions. dsRNA was synthesised using a Megascript® T7 Transcription (Ambion) kit, with a 16 h 37^o^c incubation, following manufacturer’s instructions. The dsRNA was then cleaned using a MegaClear® Transcription Clear Up (Ambion) kit, with DEPC water, twice heated at 65^o^c for 10 min, to elute the sample. The resultant dsRNA product was analysed using a nanodrop spectrometer (Nanodrop Technologies, UK) and subsequently concentrated to 3 μg/μl using a vacuum centrifuge at 35^o^c. Injections were then carried out using a nanoinjector with 69 nl of product injected directly into the thorax, between the cuticle plates of the abdomen, underneath the wing. Injections were carried out on 100, three-to-five day old, presumed mated, non-blood fed females, which were immobilised on a CO_2_ block. As a control, non-endogenous *GFP* dsRNA was injected at the same amount and concentration [62].

### Microarrays

A whole-genome microarray approach was used to determine the effect of *Maf-S* knockdown on transcriptional profiles. The transcriptional profiles of *Maf-S* knockdowns were compared against a GFP injected control. RNA was extracted from three biological replicates for each of *Maf-S(2)* injected and GFP injected controls. Mosquitoes were collected 72 h post injection, between the hours of 8 am and 2 pm. Each replicate was added to extraction buffer from the PicoPure RNA extraction kit, heated for 30 min at 42^o^c and frozen at -80^o^c as per manufacturer’s instructions. Each biological replicate for each treatment consisted of RNA, extracted using PicoPure RNA Isolation kit (Arcturus), from 7 to 12 three-five day old non-blood fed, presumed mated females. The quantity and quality of the RNA was assessed using a nanodrop spectrophotometer (Nanodrop Technologies UK) and Bioanalyser (Agilent) respectively. 100 ng of RNA was amplified and labelled with Cy3 and Cy5, using the Two colour low input Quick Amp labelling kit (Agilent) following the manufacturer’s instructions. Samples were then purified (Qiagen) with the cRNA yield and quality assessed using the nano-drop and Bioanalyser respectively. RNA from each *Maf-S* injection replicate was competitively hybridised with the GFP injected control replicates. Dye swaps were performed on each of the technical replicates for each array, to correct for dye bias. Labelled cRNAs were hybridised to the whole genome 8x15k *Anopheles gambiae* array (ArrayExpress accession number A-MEXP-2211). Microarray hybridisation, washing and scanning were performed according to previously described protocols [23]. The experiment was submitted to ArrayExpress, accession E-MTAB-4042.

### qPCR

qPCR was performed on total RNA extracted from post knockdown Tiassalé cDNA 3 days after injection using the following transcripts: AGAP008212-RA, AGAP004382-RA, AGAP008358-RA, AGAP002198-RA, AGAP008685-RA, AGAP008684-RA, AGAP004164-RA and AGAP007504-RA. RNA (4 μg) from each biological replicate (*n* = 3 per treatment group) was reverse transcribed using Oligo dT (Invitrogen) and Superscript III (Invitrogen) according to manufacturer’s instructions, DNase I (Qiagen) was applied to the column for 15 min at room temperature to remove any gDNA contamination. Quantitative real-time PCR was performed using SYBR Green Supermix III (Applied Biosystems) using an MX3005 and the associated MxPro software (Agilent). Primer Blast (NCBI) [63] was used to design primer pairs (Additional file 11: Table S6). Primers were designed to span an exon junction, where possible. Each 20 μl reaction contained 10 μl SYBR Green Supermix, 0.3 μM of each primer and 1 μl of 1:10 diluted cDNA. Standard curves were produced using whole Tiassalé cDNA, in 1, 1:5, 1:25, 1:125 dilutions. qPCR was performed with the following conditions: 3 min at 95 °C, with 40 cycles of 10 s at 95 °C and 10 s at 60 °C. All amplification efficiencies of designed primers were within acceptable range (90–120%), following MIQE guidelines [64]. Data was analysed using the ∆∆ct method [65], with GFP injected control as the comparator. All data were normalised against two housekeeping genes: S7 and EF (Additional file 11: Table S6). Welch’s t-tests were performed on the Δct values, with significance of *p* ≤ 0.05

### Bioassays

72-h post injection, a minimum of 75 female mosquitoes were assayed using WHO bioassay tube test kits [39] (minimum of 3 biological replicates of 25 mosquitoes). These assays were used under standard conditions for one-hour exposures to 0.05% deltamethrin, 0.75% permethrin, 4% DDT, 0.01% bendiocarb and 5 min exposures to 5% malathion impregnated papers. Each exposure had a corresponding untreated control of 25 female mosquitoes. Post-exposure, mosquitoes were left in a control tube, under insectary conditions for 24 h and mortality recorded. Significance tests were carried out using ANOVA with a Tukey post hoc test. Homogeneity of variance and normality of data were checked using a Bartlett test and a Shapiro Wilk test respectively; all data was transformed using an arcsine transformation.

## Additional files

Additional file 1: Figure S1. Details of the genes encoding *Maf-S cnc and Keap1* in *An gambiae*. (DOCX 37 kb)

Additional file 2: Table S1. Information on microarray data sets from insecticide resistant populations. Population name represents the area from which the strain was sampled in addition to insecticide exposure, where relevant. Country of origin, species, susceptible reference strain and paper reference are also given. (XLSX 10 kb)

Additional file 3: Figure S2. *Maf-S* co-correlated transcripts. Log_2_ fold change (y) of *Maf-S* and co-correlated transcripts across the 27 microarray studies (x axis labels) described in Additional file 2: Table S1. (PNG 37 kb)

Additional file 4: Table S2. Co-correlated transcripts for each of *cnc*, *Maf-S* and *Keap1*. Gene ID and associated description for all co-correlated transcripts (±0.8) for each gene in the *Maf-S-cnc-Keap1* pathway. Correlation was computed via a correlation matrix in R using the microarray datasets in Additional file 2: Table S1. (XLSX 19 kb)

Additional file 5: Figure S3. dsRNA knockdown levels. (a) Relative transcript levels in dsRNA *Maf-S *injected mosquitoes compared to GFP-injected and uninjected controls following injection of two alternative *Maf-S* dsRNA constructs, *Maf-S*(1) and *Maf-S*(2). (b) Relative transcript levels for each splice variant (*Maf-S*-RA: AGAP010405-RA (RA) and *Maf-S*-RB: AGAP010405-RB (RB)) following injection of the *Maf-S*(2) dsRNA construct. For details of primers see Additional file 11: Table S6. Error bars represent the standard error of the mean. (JPEG 37 kb)

Additional file 6: Figure S4. Anti-oxidant response element motif and corresponding presence/absence in up- and down-stream regions of *Maf-S* co-correlated transcripts. (a) JASPAR Core Insect motif representing the anti-oxidant- or xenobiotic- response element. (b) Presence of the motif 2000 bp up- and down-stream of *Maf-S* co-correlated transcripts, showing transcript ID, region of motif location and the representative motif. (PNG 78 kb)

Additional file 7: Table S3. Significant transcripts on *Maf-S-RNAi microarray* data set. Transcript ID, log_2_ fold change, adjusted *p*-value and B-value as calculated by limma analysis for all significant transcripts on the arrays comparing *Maf-S* silenced to GFP-injected controls. (XLSX 198 kb)

Additional file 8: Figure S5. Volcano plot showing transcript levels in *Maf-S* knockdowns compared to GFP controls. Significantly differentially expressed probes are shown in black (adjusted *p* ≤ 0.05), detoxification family members are shown in shapes indicated on the key (ABC = ABC transporter, COE = carboxylesterase, CYP = cytochrome p450, GST = glutathione-S-transferase and UGT = (UDP-glucuronosyltransferase). Probes are labelled with associated transcript IDs/gene names where *p* ≤ 0.001. Genes down-regulated in the *Maf-S* knockdowns probes are shown to the left and up-regulated probes to the right. (PNG 88 kb)

Additional file 9: Table S4. Significant GO terms for significantly up- and down-regulated transcripts. Significant GO terms for transcript lists significantly up- (bold) or down- (italic) regulated after *Maf-S* knockdown. GO terms, Benjamini adjusted *p*-values and transcript membership shown as calculated by DAVID [66]. (XLSX 10 kb)

Additional file 10: Table S5. Detoxification transcripts significantly differentially expressed in *Maf-S* microrarray. 64 transcripts belonging to Phase I, II or III detoxification family members (glutathione transferases, cytochrome P450s, UGTs and ABCs) that show significant differential expression after knockdown of *Maf-S*. In addition to fold change from the *Maf-S* knockdown arrays data from previously published experiments comparing gene expression between Tiassalé and a susceptible population, N’Gousso to indicate which of the genes regulated by *Maf-S* are differentially expressed in the Tiassalé populations and thus candidates for conferring resistance [20]. (XLSX 12 kb)

Additional file 11: Table S6. Primer Tables. RNAi primers used in the synthesis of dsRNA, each with a T7 promoter region (shown in lower case) and sequences of primers used in all qPCR validation. (XLSX 11 kb)

## Abbreviations

ARE: Antioxidant Response Element
cDNA: Complementary DNA
COE: Carboxylesterase
cRNA: Complementary RNA
dsRNA: Double Stranded RNA
GFP: Green Fluorescent Protein
GO: Gene Ontology
GST: Glutathione-S-Transferase
IRS: Indoor residual spraying
qPCR: Quantitative PCR
RNAi: RNA Interference
UGT: UDP-glucuronosyltransferas
