# Supplementary material for: The transcription factor Maf-S regulates metabolic resistance to insecticides in the malaria vector Anopheles gambiae
Source: BMC Genomics. 2017 Aug 30;18:669. doi: 10.1186/s12864-017-4086-7 (PMC5577768; doi:10.1186/s12864-017-4086-7)
Supplement: Supplementary file 1 — Details of the genes encoding Maf-S cnc and Keap1 in An gambiae. (DOCX 37 kb) [file 12864_2017_4086_MOESM1_ESM.docx]

Additional File 1:

*The Maf-S-cnc-Keap1 pathway in* Anopheles gambiae

A single ortholog of the *Drosophila Maf-S (CG9954)* is present in the genome of *An. gambiae* on chromosome arm 3L. Anopheles gambiae Maf-S has two splice variants: AGAP010405-RA contains two coding exons and encodes a putative protein of 146 amino acids; AGAP010405-RB encodes a putative protein of 136 amino acids encoded by a single exon (Figure S1). Both splice variants contain the bZip-Maf domain which is a basic leucine zipper motif, allowing dimerization and DNA binding [1].

*Keap1* is present in *An. gambiae* in a two-to-one homology relationship with *D. melanogaster*; both paralogs (AGAP003645 (1014 amino acids) and AGAP012550 (256 amino acids)) are annotated as Kelch-like proteins showing relatively low query coverage (<40%) but high identity when compared to *D. melanogaster* *Keap1* (*CG3962*) of 73% and 75%, indicative of shared functional domains. The two homologs of *Keap1* present in *Anopheles* were manually examined using VectorBase for the presence of both a BTB/POZ domain and the 6 kelch repeats necessary for binding to *cnc* [2]. Only AGAP003645 contained all these features with AGAP012550 representing a truncated version of AGAP003645, lacking the kelch repeats (Figure S1). Thus, AGAP003645 is therefore the probable functional ortholog of Keap1.

The single ortholog of *Drosophila cnc* found in *An. gambiae* (AGAP005300) is predicted to have three isoforms: *cncA, cncB* and *cncC*, all splice variants contain the bZIP motif necessary for binding *Maf-*S; however, only *cncA* and *cncB* contain an ETGE motif thought necessary to bind *Keap1* [3] (Figure S1).

Figure S1: **Schematic of *Maf-S* *cnc and Keap1* in *An gambiae*.** The two splice variants of *Maf-S* (AGAP010405) are shown with the leucine-zipper motif represented in green. Splice sites are shown by dashed black lines. The two *Keap1* homologs (AGAP003645 and AGAP012550) are shown with the BTB/POZ and Kelch domains represented in red and blue respectively. The presence of both these domains are necessary for *cnc* binding [4]. The three *cnc* (AGAP005300) splice variants *cncA, cncB* and *cncC* are illustrated with the b-zip domain and the ETGE motif in yellow and black respectively. Again, the presence of both are necessary for *Maf-S* binding [5].


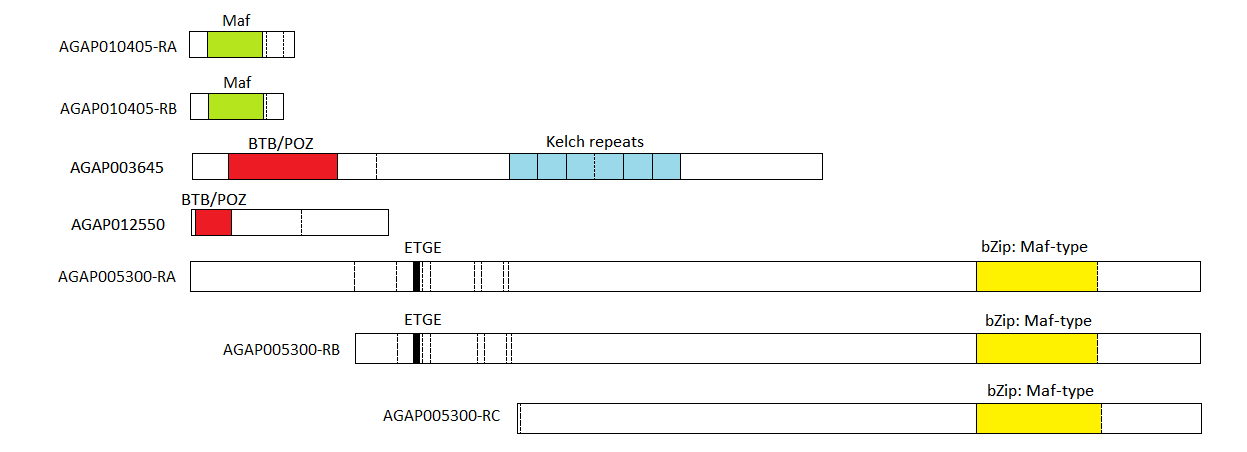


References

1. Katsuoka F, Yamamoto M. Small Maf proteins (MafF, MafG, MafK): History, structure and function. Gene. 2016;586:197–205.

2. Sykiotis GP, Bohmann D. Keap1/Nrf2 signaling regulates oxidative stress tolerance and lifespan in Drosophila. Dev. Cell. Elsevier; 2008;14:76–85.

3. Kobayashi M, Itoh K, Suzuki T, Osanai H, Nishikawa K, Katoh Y, et al. Identification of the interactive interface and phylogenic conservation of the Nrf2‐Keap1 system. Genes to Cells. Wiley Online Library; 2002;7:807–20.

4. Zipper LM, Mulcahy RT. The Keap1 BTB/POZ dimerization function is required to sequester Nrf2 in cytoplasm. J. Biol. Chem. ASBMB; 2002;277:36544–52.

5. Tong KI, Katoh Y, Kusunoki H, Itoh K, Tanaka T, Yamamoto M. Keap1 recruits Neh2 through binding to ETGE and DLG motifs: characterization of the two-site molecular recognition model. Mol. Cell. Biol. Am Soc Microbiol; 2006;26:2887–900.
