# Supplementary figures and images for: The transcription factor Maf-S regulates metabolic resistance to insecticides in the malaria vector Anopheles gambiae
Source: BMC Genomics. 2017 Aug 30;18:669. doi: 10.1186/s12864-017-4086-7 (PMC5577768; doi:10.1186/s12864-017-4086-7)

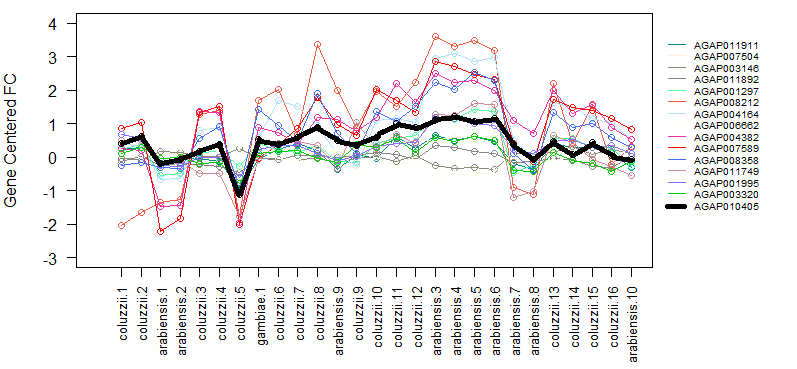

Supplement: Supplementary file 3 — Maf-S co-correlated transcripts. Log2 fold change (y) of Maf-S and co-correlated transcripts across the 27 microarray studies (x axis labels) described in Additional file 2: Table S1. (PNG 37 kb) [file 12864_2017_4086_MOESM3_ESM.png]

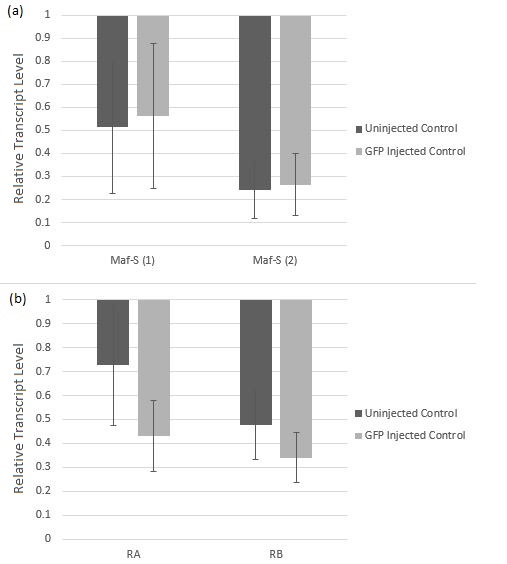

Supplement: Supplementary file 5 — dsRNA knockdown levels. (a) Relative transcript levels in dsRNA Maf-S injected mosquitoes compared to GFP-injected and uninjected controls following injection of two alternative Maf-S dsRNA constructs, Maf-S(1) and Maf-S(2). (b) Relative transcript levels for each splice variant (Maf-S-RA: AGAP010405-RA (RA) and Maf-S-RB: AGAP010405-RB (RB)) following injection of the Maf-S(2) dsRNA construct. For details of primers see Additional file 11: Table S6. Error bars represent the standard error of the mean. (JPEG 37 kb) [file 12864_2017_4086_MOESM5_ESM.jpg]

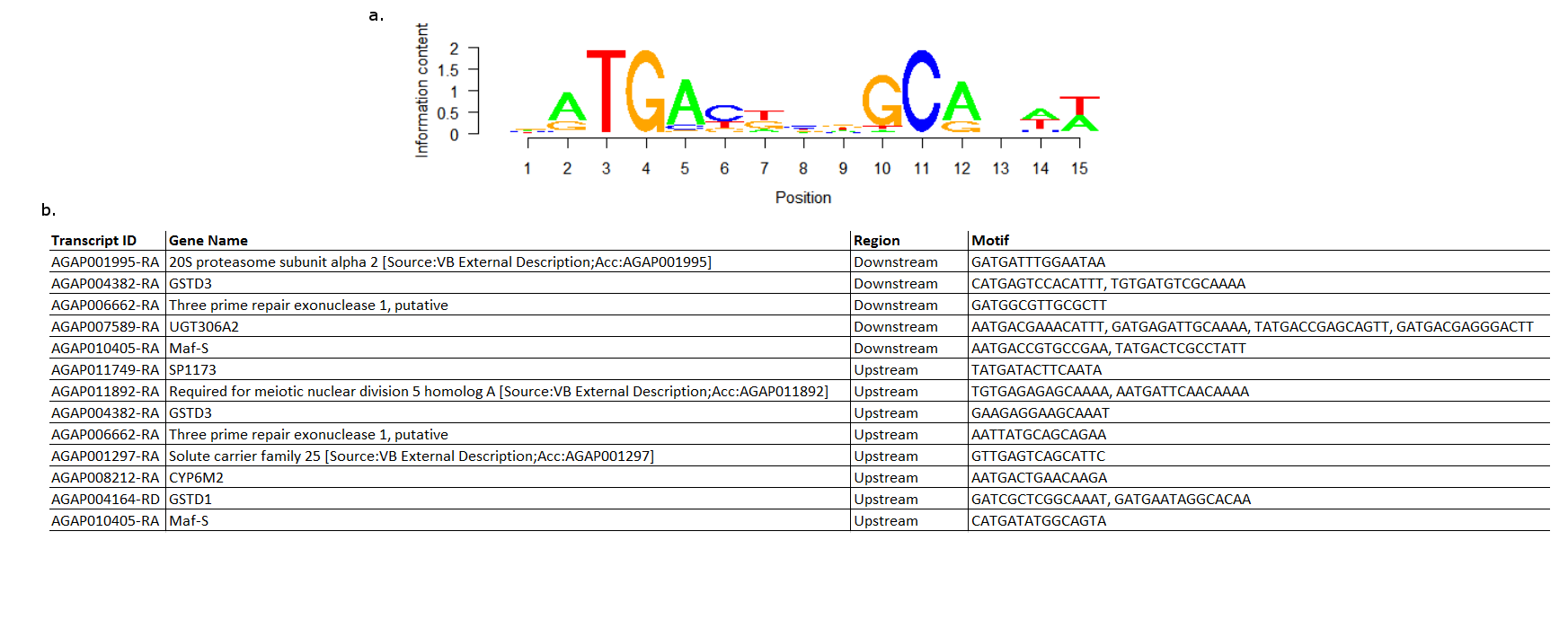

Supplement: Supplementary file 6 — Anti-oxidant response element motif and corresponding presence/absence in up- and down-stream regions of Maf-S co-correlated transcripts. (a) JASPAR Core Insect motif representing the anti-oxidant- or xenobiotic- response element. (b) Presence of the motif 2000 bp up- and down-stream of Maf-S co-correlated transcripts, showing transcript ID, region of motif location and the representative motif. (PNG 78 kb) [file 12864_2017_4086_MOESM6_ESM.png]

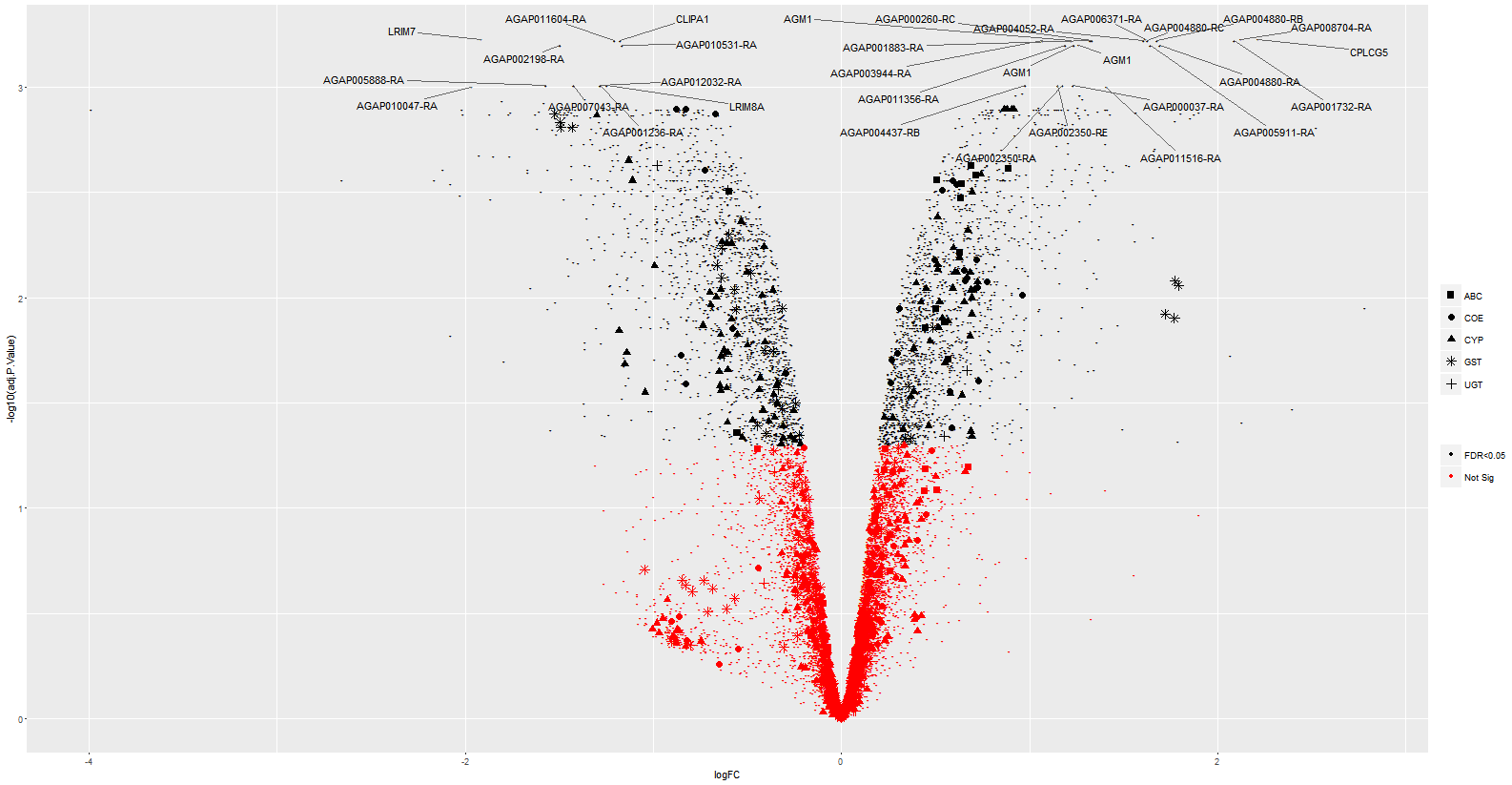

Supplement: Supplementary file 8 — Volcano plot showing transcript levels in Maf-S knockdowns compared to GFP controls. Significantly differentially expressed probes are shown in black (adjusted p ≤ 0.05), detoxification family members are shown in shapes indicated on the key (ABC = ABC transporter, COE = carboxylesterase, CYP = cytochrome p450, GST = glutathione-S-transferase and UGT = (UDP-glucuronosyltransferase). Probes are labelled with associated transcript IDs/gene names where p ≤ 0.001. Genes down-regulated in the Maf-S knockdowns probes are shown to the left and up-regulated probes to the right. (PNG 88 kb) [file 12864_2017_4086_MOESM8_ESM.png]
